# Benefits of alanyl-glutamine and omega-3 PUDAs in postoperative gastroduodenal perforation patients: A single-center retrospective study

**DOI:** 10.1097/MD.0000000000042186

**Published:** 2025-05-23

**Authors:** Xuanjun Liu, Weixu Mao, Guowei Zhao, Qigang Li, Juan Liao, Gan He

**Affiliations:** a Department of Gastrointestinal Surgery, Yongchuan Hospital of Chongqing Medical University, Yongchuan, Chongqing, China; b Department of Respiratory Medicine, The Affiliated Yongchuan District Traditional Chinese Medicine Hospital of Chongqing Medical University, Yongchuan District, Chongqing, China; c Central Laboratory, Yongchuan Hospital of Chongqing Medical University, Yongchuan, Chongqing, China.

**Keywords:** alanyl-glutamine, gastroduodenal perforation, inflammation, nutritional status, omega-3 polyunsaturated fatty acids, parenteral nutrition

## Abstract

This clinical study combined alanyl-glutamine and omega-3 polyunsaturated fatty acids (ω-3 PUFAs) to investigate the effects of parenteral nutrition on postoperative inflammation and nutritional status in patients with gastroduodenal perforation to provide a basis and support for the use of clinical immunonutrients. Patients with gastroduodenal perforations who underwent surgery between January 2018 and December 2023 were included. From the first to the seventh postoperative day, Group A (GA) received conventional postoperative nutrition with fat emulsion (20%), amino acids (17), and glucose (11%) injection; Group B (GB), building on GA’s regimen, was additionally treated with 10 g/day of ω-3 PUFAs; and Group C (GC), expanding on GB’s regimen, was additionally treated with 10 g/day of alanyl-glutamine. A total of 168 patients were included in the study, with 71 in GA, 30 in GB, and 67 in GC. Total protein and albumin (Alb) levels increased in all 3 groups, with GC showing a more significant increase compared to GB and GA (TP: 7.73 ± 5.00 vs 4.35 ± 5.85 vs 3.92 ± 5.07, *P* < .05; Alb: 4.07 ± 4.52 vs 1.79 ± 4.00 vs 2.11 ± 4.10, *P* < .05); C-reactive protein levels decreased in all 3 groups, with the most pronounced decrease in GC (93.71 ± 80.97 vs 72.04 ± 80.48 vs 55.79 ± 83.68, *P* < .05); the length of hospitalization and among the 3 groups was statistically significant (10.7 ± 2.27 vs 13.39 ± 4.66 vs 12.52 ± 3.46, *P* < .05), and GB was shorter than GA; the incidence of postoperative complications was significantly lower in GC than in other groups (*P* < .05). Parenteral nutrition supplemented with alanyl-glutamine and ω-3 PUFAs can increase postoperative total protein and Alb levels, thereby improving patient nutritional status, reducing the production of the inflammatory marker C-reactive protein, mitigating the inflammatory response, and decreasing the incidence of postoperative complications, thus improving patient prognosis.

## 1. Introduction

The complications of peptic ulcer disease include perforation, bleeding, and obstruction. Gastroduodenal perforation, although less common than bleeding (approximately 1:6 ratio), is one of the most frequent indications for emergency surgery.^[1]^ The clinical presentation of gastroduodenal perforation typically features sudden onset of abdominal pain. Localized or generalized peritonitis is characteristic of perforated peptic ulcers.^[2]^ In severe cases, the blood pressure decreases, leading to shock and even death. Surgery is the main treatment for gastroduodenal perforation. However, surgical stress, postoperative complications, postoperative fasting, and poor immune function pose major challenges to patient recovery and survival.^[3]^ The immune system plays a key role in preventing infections and clearing pathogens. 2021 ESPEN practical guideline: clinical nutrition in surgery recommends the use of appropriate perioperative and postoperative nutritional supportive therapy for critical and major surgical patients at risk for malnutrition.^[4]^

Immunonutrients such as omega-3 polyunsaturated fatty acids (ω-3 PUFAs), glutamine, and arginine play crucial roles in enhancing immune function, reducing inflammatory responses, and promoting tissue recovery.^[5,6]^ ω-3 PUFAs are essential fatty acids that include eicosapentaenoic acid, docosahexaenoic acid, and alpha-linolenic acid.^[7]^ ω-3 PUFAs have excellent anti-inflammatory effects and can be used to improve the immune function and nutritional status of patients.^[8]^ Alanyl-glutamine, typically considered a nonessential amino acid, is conditionally essential during periods of stress such as surgery, critical illness, and stress. It plays a role in the synthesis of various amino acids, nucleic acids, and proteins and may modulate the body’s immune response.^[9]^ Therefore, postoperative patients often receive ω-3 PUFAs, glutamine, and other forms of nutritional support to promote rapid recovery, and their clinical effects are well-recognized. Nevertheless, evidence from some studies indicates that immunonutrition may not enhance patient outcomes and may increase the risk of mortality.^[10–12]^ Concurrently, the increase in immunonutrition formulas, including ω-3 PUFAs, arginine, and RNA, has highlighted their benefits. ESPEN practical guideline recommend the postoperative administration of a specific formula enriched with (arginine, omega-3 fatty acids, and ribonucleotides) should be administered to malnourished patients undergoing major cancer surgery.^[4]^ However, the optimal formula, dosage, route of administration, and duration of immunonutrition remains debatable.

To investigate the effects of parenteral nutrition (PN) supplemented with alanyl-glutamine and ω-3 PUFAs on the postoperative inflammation and nutritional status of patients with gastroduodenal perforation, 168 patients who underwent gastroduodenal perforation surgery were retrospectively studied.

## 2. Information and methods

### 2.1. General information

This retrospective study evaluated patients who underwent surgery for gastroduodenal perforation at Yongchuan Hospital of Chongqing Medical University between January 2018 and December 2023. All patients participating in this study signed an informed consent form, and all methods were performed in accordance with relevant guidelines and regulations. This study was approved by the local ethics committee (protocol no. 2024LLS008). A total of 168 patients who underwent surgery for gastroduodenal perforation were identified as the study population based on the inclusion and exclusion criteria.

The inclusion criteria were as follows: (1) age ≥ 18 years; (2) diagnosis of gastroduodenal perforation (preoperative diagnosis of perforation or intraoperative confirmation of perforation); (3) undergoing gastroduodenal perforation repair with complete clinical data and clear and uncontested relevant indexes; (4) no history of other surgical treatments of the abdomen; (5) no history of mental disorder, with judgmental, receptive, and cognitive abilities, and with normal verbal communication; and (6) adopting the surgical criteria of (a) a typical clinical manifestation of acute gastroduodenal ulcer perforation; (b) radiological auxiliary examination to support the diagnosis, with visible free gas under the diaphragm; (c) surgical confirmation of perforation, severe intra-abdominal infection, and verified edema, with a large amount of purulent exudate; (d) no bleeding or obstruction complications, or other systemic organic diseases that cannot tolerate surgery.

The exclusion criteria were as follows: (1) combined gastrointestinal system diseases other than gastroduodenal ulcer; (2) serious dysfunction of parenchymal organs; (3) coagulation dysfunction; (4) combined serious internal environmental dysfunction; (5) abnormalities in mental status and cognitive function; (6) other serious systemic diseases; (7) accompanying gastrointestinal hemorrhage; and (8) refusal to undergo surgery or transfer to another hospital for treatment due to irresistible factors.

### 2.2. Methods

From the first to the seventh day postoperatively, each patient in all 3 groups received 0.15 to 0.30 g nitrogen/kg each day in PN solution by using peripheral venous catheterization. The ratio of glucose to lipids in the PN solution was 1 to 1.5:1. Group A (GA) only utilized the fat emulsion (20%), amino acids (17), and glucose (11%) injection, with no ω-3 PUFAs in this fat emulsion and no alanyl-glutamine in the 17 amino acid components. Group B (GB), built on the GA regimen, received 10 g of ω-3 PUFAs. In Group C (GC), 10 g alanyl-glutamine was added to the GB regimen. (Table 1) (see Table S1, Supplemental Digital Content, https://links.lww.com/MD/P7, which illustrates the PN composition of parenteral nutrition).

**Table 1:** Composition of parenteral nutrition.

Nutrient	Dosage		
	GA (n = 71)	GB (n = 30)	GC (n = 67)
Fat emulsion,* g/kg	1.0–2.0	1.0–2.0	1.0–2.0
Amino acids,* g/kg	1.0–2.0	1.0–2.0	1.0–2.0
Glucose,* g/kg	2.0–6.0	2.0–6.0	2.0–6.0
Multivitamins,† mL	5	5	5
Multiple microelements (II),‡ mL	10	10	10
ω-3 PUFAs,§ g	0	10	10
Alanyl-glutamine,∥g	0	0	10

* Fat emulsion (20%), amino acids (17), and glucose (11%) injection (Fresenius Kabi, Germany); no ω-3 PUFAs in this fat emulsion; no alanyl-glutamine in the 17 amino acid components.

† Multivitamins for injection (12) (FAREVA PUA, France) contains 12 vitamins, including vitamin A, vitamin D3, vitamin E, etc.

‡ Multiple microelement for injection (II) (Fresenius Kabi, Germany) can meet the daily needs of 9 trace elements, such as chromium, copper, and iron.

§ω -3 fish oil fat emulsion injection (Fresenius Kabi, Germany) containing 10 g of refined fish oil and 1.2 g of lecithin (100 mL).

∥ Alanyl-glutamine injection (Fresenius Kabi, Germany) containing 10 g alanyl-glutamine.

### 2.3. Observations and test indicators

Data on laboratory measurements and clinical outcome were collected by reviewing the medical record.

#### 2.3.1. Baseline data

Statistical baseline data on patient age, sex, location of perforation, comorbid underlying disease, and preoperative blood results (T1).

#### 2.3.2. Observation of clinical indexes

Observation and count of patients’ operation time, ICU hospitalization time, antibiotic use time, human albumin (Alb) supplementation dose, and hospitalization cost.

#### 2.3.3. Nutritional, immunological, and inflammatory indices

Blood was drawn from the patient’s vein before administration of PN (U1) and on day 5 of administration (U2), and the levels of hemoglobin, total protein, Alb, white blood cell, lymphocytes, and C-reactive protein (CRP) were measured.

#### 2.3.4. Postoperative complications and incidence

Observe and count the occurrence of postoperative complications (including incision infection, abdominal infection, lung infection, urinary tract infection, bloodstream infection, digestive bleeding, venous thrombosis, and pulmonary embolism) and incidence of postoperative complications.

### 2.4. Statistical methods

SPSS 26.0 software was used for the statistical analysis of the study results. Measurement data are expressed as mean ± SD or median (P_25_, P_75_), and count data are expressed as frequency and percentage (n [%]). Based on the normality of the data distribution, we used one-way ANOVA or the Kruskal–Wallis test for comparisons among the 3 groups, and Fisher least significant difference (LSD) for pairwise comparisons in the post hoc analysis. For within-group comparisons between U2 and U1, we employed the paired *t* test or Wilcoxon Mann–Whitney test. Additionally, the *χ*^2^ test or Fisher exact test was used to compare the group ratios, with a statistically significant difference defined as *P* < .05.

## 3. Results

### 3.1. Patients

**Figure 1.**
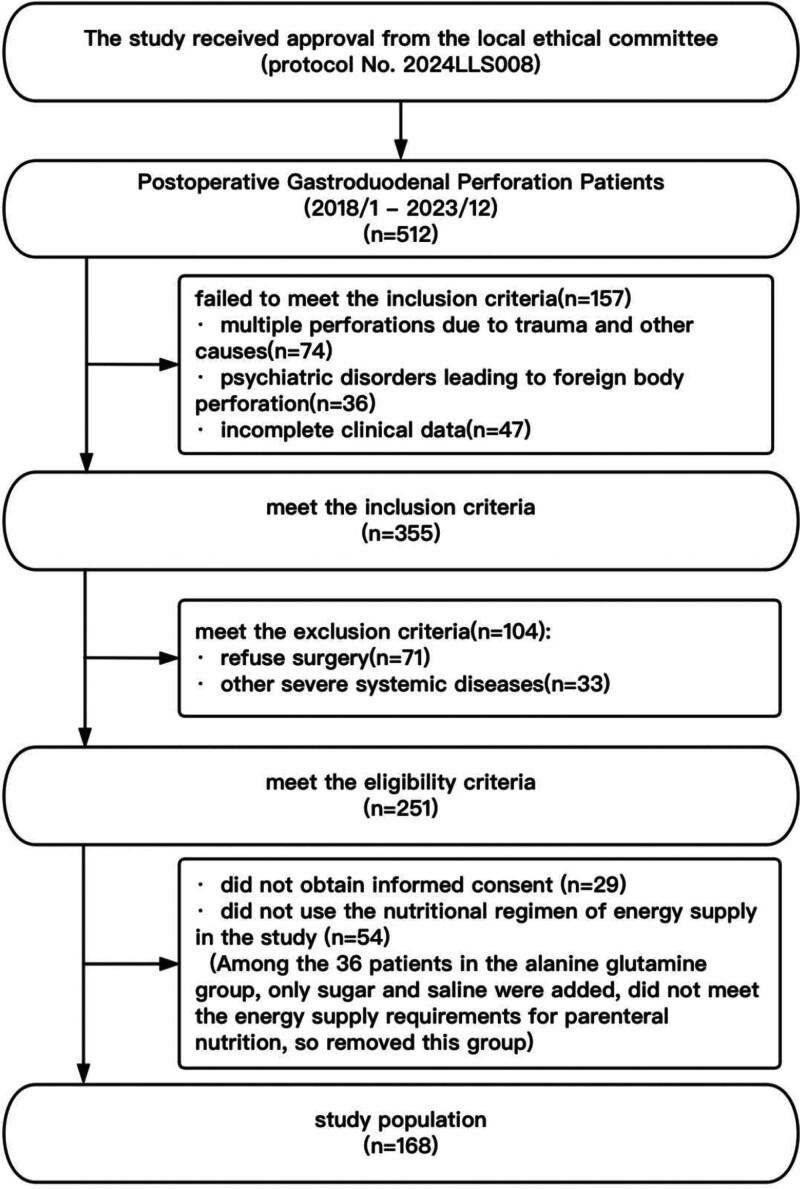
Flow chart of this retrospective study.

A total of 512 patients with gastroduodenal perforation were identified by the hospital system during the data collection process. After applying the inclusion and exclusion criteria, 251 participants were screened. Later, 29 individuals were unwilling to disclose their clinical information, and 54 did not receive nutritional support per energy requirement (all the alanyl glutamine groups used nutritional support by adding sugar saline, which did not satisfy the study, and the group was considered and deleted). Ultimately, this retrospective study included 168 participants (133 males and 35 females (see Fig. 1, illustrating the flow chart of this study).

### 3.2. Comparison of baseline data of the 3 groups of patients

When comparing admission blood test results (T1) and the baseline characteristics of the 3 groups, including age, sex, perforation site, perforation location, and underlying diseases, there were no statistically significant differences (*P* > .05) (see Table 2, which illustrates the patients’ baseline information).

**Table 2:** Comparison of patients’ baseline information.

Baseline information	GA (n = 71)	GB (n = 30)	GC (n = 67)	F/H/*χ*^2^	*P*
Age (in years)	63.66 ± 16.19	66.87 ± 12.48	66.78 ± 13.55	0.955	.387
Sex [cases (%)]					
Male	61 (85.9%)	20 (66.7%)	52 (77.6%)	4.901	.086
Female	10 (14.1%)	10 (33.3%)	15 (22.4%)		
Perforation location [cases (%)]					
Gastric	51 (71.8%)	20 (66.7%)	44 (65.7%)	0.66	.719
Duodenum	20 (28.8%)	10 (33.3%)	23 (34.3%)		
Underlying diseases [cases (%)]					
Hypertension	22 (31.0%)	8 (26.7%)	19 (28.4%)	0.226	.893
Diabetes	4 (5.6%)	2 (6.7%)	7 (10.4%)	1.178	.555
Coronary heart disease	6 (8.5%)	2 (6.7%)	7 (10.4%)	0.399	.819
Alb (g/L)	36.9 ± 6.8	37.2 ± 5.1	36.8 ± 5.1	0.031	.970
TP (g/L)	65.5 ± 10.9	65.4 ± 8.5	66.2 ± 7.4	0.119	.888
HB (g/L)	123.1 ± 25.7	122.3 ± 27.9	124.7 ± 28.4	0.097	.907
CRP (mg/L)	54.8 (6128.4)	34.1 (2.83, 87.7)	20.3 (3.6103.7)	2.053	.358
WBC (10^9^/L)	12.1 ± 6.6	12.1 ± 5.3	11.2 ± 5.7	0.455	.635
Lym (10^9^/L)	0.8 ± 0.5	0.6 ± 0.3	0.6 ± 0.4	2.827	.062

Alb = albumin, CRP = C-reactive protein, GA = Group A, GB = Group B, GC = Group C, HB = hemoglobin, Lym = lymphocytes, TP = total protein, WBC = white blood cell.

### 3.3. Comparison of nutritional, immunological, and inflammatory indicators

**Figure 2.**
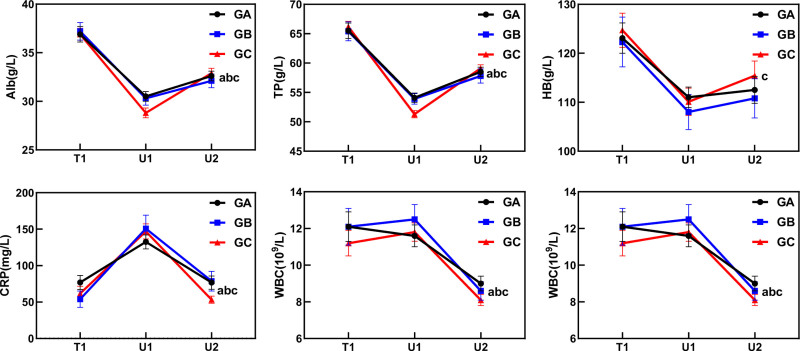
Plot of changes in blood tests of 3 groups. (A) Difference exists between U2 and U1 in GA (*P* < .05). (B) Difference between U2 and U1 in GB (*P* < .05). (C) Difference between U2 and U1 in GC (*P* < .05).

Blood test results were compared among the 3 groups: prior to PN initiation (U1), on the fifth day post-PN (U2), and the changes between the 2 time points (ΔU2-U1). In U1, the data revealed that the differences in ALB and TP levels among the 3 groups were statistically significant (*P* < .05). Post hoc LSD analysis revealed that GC had significantly lower TP levels than GA and GB (GC < GB < GA), and significantly lower ALB levels than GA, while no significant differences were observed in the remaining data. At U2, there were no significant differences in the data of the 3 groups. In the adjusted U2 values derived from U1 as the baseline for correction, the data revealed that the differences in ALB and TP levels among the 3 groups were statistically significant (*P* < .05). Post hoc LSD analysis revealed that GC had significantly lower TP levels than GA and GB (GC < GB < GA), and significantly lower ALB levels than GA. In the ΔU2-U1 analysis, changes in CRP, TP, and Alb levels were significantly different (*P* < .05). Post hoc LSD analysis revealed that GC had significantly higher TP and Alb levels than GA and GB (GC < GB < GA), and significantly higher CRP levels than GA. No significant differences were observed in any of the remaining data (see Table 3 and Fig. 2, which illustrates patients’ nutritional, immunological, and inflammatory indicators).

**Table 3:** Comparison of nutritional, immunological, and inflammatory indicators in patients.

		GA (n = 71)	GB (n = 30)	GC (n = 67)	F/H	*P*
ALB (g/L)	U1	30.5 ± 4.6	30.3 ± 3.8	28.8 ± 3.8†	3.201	.043*
	U2	32.6 ± 4.4‡	32.1 ± 3.8‡	32.9 ± 3.8‡	0.399	.672
	ΔU2-U1	2.11 ± 4.10	1.79 ± 4.00	4.07 ± 4.52†§	4.776	.010*
	Adjust U2	33.0 ± 1.9	32.9 ± 1.6	32.2 ± 1.6†	3.201	.043*
TP (g/L)	U1	54.1 ± 6.6	53.9 ± 4.9	51.3 ± 4.6†§	4.832	.009*
	U2	58.5 ± 6.7‡	57.8 ± 6.8‡	59.1 ± 5.2‡	0.444	.642
	ΔU2-U1	4.35 ± 5.85	3.92 ± 5.07	7.73 ± 5.00†§	8.600	.000*
	Adjust U2	59.3 ± 3.9	59.1 ± 2.9	58.6 ± 3.4†§	4.832	.009*
HB (g/L)	U1	111 ± 17.9	108 ± 19.9	110.1 ± 22.5	0.226	.798
	U2	112.5 ± 22.7‡	110.8 ± 21.8‡	115.4 ± 24.4‡	0.475	.623
	ΔU2-U1	1.56 ± 12.87	2.83 ± 9.27	5.25 ± 11.01	1.779	.172
	Adjust U2	114.2 ± 17.9	111.3 ± 19.8	113.4 ± 22.4	0.226	.798
CRP (mg/L)	U1	124.9 (85.5, 157.7)	141.8 (61.5, 228.8)	145.7 (72.3, 188.9)	1.338	.512
	U2	62.3 (18.0, 110.6)∥	59.2 (19.7, 123.8)∥	42.0 (19.7, 82.6)∥	1.434	.488
	ΔU2-U1	55.79 ± 83.68	72.04 ± 80.48	93.71 ± 80.97†	3.689	.027*
	Adjust U2	64.9 ± 25.6	70.6 ± 31.9	69.4 ± 26.7	0.687	.505
WBC (10^9^/L)	U1	11.6 ± 4.7	12.5 ± 4.2	11.8 ± 4.4	0.399	.671
	U2	9.0 ± 3.5‡	8.6 ± 2.9‡	8.1 ± 2.6‡	1.569	.211
	ΔU2-U1	2.63 ± 5.05	3.84 ± 4.02	3.76 ± 3.67	1.452	.237
	Adjust U2	8.5 ± 1.2	8.7 ± 1.1	8.5 ± 1.2	0.399	.671
Lym (10^9^/L)	U1	0.7 ± 0.4	0.6 ± 0.3	0.7 ± 0.4	0.690	.503
	U2	1.0 ± 0.4‡	1.1 ± 0.5‡	1.1 ± 0.5‡	0.139	.871
	ΔU2-U1	0.30 ± 0.36	0.45 ± 0.38	0.35 ± 0.43	1.517	.222
	Adjust U2	1.1 ± 0.3	1.0 ± 0.2	1.1 ± 0.3	0.690	.503

Alb = albumin, CRP = C-reactive protein, GA = Group A, GB = Group B, GC = Group C, HB = hemoglobin, Lym = lymphocytes, TP = total protein, WBC = white blood cell.

* Significant difference among the 3 groups at *P* < .05, as determined by one-way ANOVA.

† Significant difference between GC and GA at *P* < .05.

‡ Significant difference between U2 and U1 at *P* < .05, as determined by the paired *t* test.

§ Significant difference between GC and GB at *P* < .05, as determined by LSD.

∥ Significant difference between U2 and U1 at *P* < .05, as determined by the Wilcoxon Mann–Whitney test.

### 3.4. Comparison of patients’ clinical data

Analysis of surgical time, length of ICU stay, duration of antibiotic use, Alb supplementation, hospitalization fees, and length of hospitalization among the 3 groups showed that the length of hospitalization was significantly different (*P* < .05), whereas GB had a shorter stay than GA. However, no other significant differences were observed among the 3 groups for the remaining parameters (*P* > .05) (see Table 4, illustrating the patients’ clinical data).

**Table 4:** Comparison of patients’ clinical data.

	GA (n = 71)	GB (n = 30)	GC (n = 67)	F	*P*
Surgical time (h)	109.99 ± 56.68	95.83 ± 45.9	101.52 ± 39.35	1.053	.351
Length of ICU stay (h)	0 (0, 33.0)	4.5 (0, 15.0)	0 (0, 16.5)	2.786	.248
Duration of antibiotic use (d)	12.04 ± 3.39	11.77 ± 5.62	11.76 ± 2.82	0.118	.889
Albumin supplementation (vial)	5.34 ± 5.72	4.1 ± 5.98	4.37 ± 4.33	0.843	.432
Hospital fees (1000 RMB)	31.1 ± 12.5	29.0 ± 10.5	31.0 ± 12.3	0.357	.700
Length of hospitalization (d)	13.39 ± 4.66	10.7 ± 2.27†	12.52 ± 3.46	5.133	.007*

* Significant differences among the 3 groups at *P* < .05.

† Statistically significant at *P* < .05, compared to GA.

### 3.5. Postoperative complications and complication rate

We analyzed the incidence of postoperative complications across the groups, including incision infection, lung infection, gastrointestinal hemorrhage, and pulmonary embolism, excluding duplicate cases. The incidence rate of postoperative complications was significantly lower than in the GA and GB groups (*P* < .05). However, no significant differences were found in the individual comparisons of complications, such as incision infection, abdominal infection, lung infection, urinary tract infection, bloodstream infection, gastrointestinal bleeding, venous thrombosis, and pulmonary embolism among the 3 groups (see Table 5, illustrating patient complications).

**Table 5:** Comparison of postoperative complications and incidence of postoperative complications in patients.

	GA (n = 71)	GB (n = 30)	GC (n = 67)	*χ* ^2^	*P*
Cutaneous infection	1 (1.4%)	1 (3.3%)	0	2.235	.326
Abdominal infection	2 (2.8%)	1 (3.3%)	1 (1.5%)	0.937	.672
Lung infection	8 (11.3%)	2 (3.7%)	4 (6.0%)	1.399	.497
Urinary tract infection	0	0	1 (1.5%)	1.678	.577
Bloodstream infection	3 (4.2%)	1 (3.3%)	1 (1.5%)	1.095	.609
Gastrointestinal bleeding	3 (4.2%)	1 (3.3%)	0	2.957	.235
Venous thrombosis	8 (11.3%)	4 (13.3%)	3 (4.5%)	2.826	.243
Pulmonary embolism	1 (1.4%)	0	0	1.562	1
Total postoperative complications	22 (31.0%)	10 (33.3%)	8 (11.9%)	8.719	.013*

* Significant difference among the 3 groups at *P* < .05.

## 4. Discussion

Gastroduodenal perforation is a critical condition with rapid onset that is relatively common in clinical practice, and surgical treatment is of great significance for the alleviation of clinical symptoms.^[2,13]^ However, the nutritional risk of gastroduodenal perforation is often due to insufficient nutritional intake caused by severe preoperative infection and prolonged postoperative fasting, and postoperative nutritional supportive therapy is often administered to promote rapid recovery.^[14]^

ω-3 fish oil fat emulsion containing ω-3 PUFAs can be decomposed to produce docosahexaenoic acid and eicosapentaenoic acid, reducing the generation of leukotriene B4 and the pro-inflammatory factor PGE2, which alleviates the inflammatory response, promotes phagocytosis of phagocytes, reduces the secretion and release of a variety of immune cells, and exerts immunomodulation.^[15–17]^ Glutamine for the body’s immune cells and gastrointestinal mucosal cells to provide nutritional support to improve their immune function; in addition, glutamine is also a class of commonly used in clinical intestinal mucosal protective agents, is a class of important nutrients for the repair of the intestinal mucosa, can improve the metabolic balance of the patient’s body, to correct the patient’s nutritional deficiencies.^[18–20]^ Clinically, glutamine is often administered either in its free form, known as an isolated amino acid, or combined with another amino acid to form a dipeptide, with alanyl-glutamine being the most recognized example.^[21]^ Therefore, for patients with gastroduodenal perforation, alanyl-glutamine can protect the gastrointestinal mucosa and provide adequate nutrition. On this basis, the combined use of ω-3 PUFAs can improve the nutritional status and immune function of patients, which is conducive to their recovery and prognosis.

A meta-analysis by Xiong et al,^[22]^ which included 26 studies involving 1678 patients, aimed to study the effect of glutamine on postoperative colorectal cancer patients and showed that glutamine significantly increased Alb and prealbumin levels and attenuated postoperative inflammatory response. Similarly, Tang et al^[23]^ reviewed colorectal cancer patients who underwent surgery within the past 7 years and found that postoperative parenteral glutamine supplementation was effective in decreasing the incidence of postoperative complications, promoting the recovery of bowel function, and increasing Alb levels in patients undergoing surgery for colorectal cancer. A meta-analysis of fat emulsions in surgical patients included 19 randomized controlled trial (RCT) reports and showed that supplementation with ω-3 PUFAs reduced the incidence of infectious complications compared to other fat emulsions.^[24]^ Similarly, the results of another meta-analysis on major abdominal surgery by He et al^[25]^ showed that postoperative patients who received ω-3 PUFAs had a lower rate of infections than the control group; however, the difference between the 2 groups in terms of inflammatory factors was not statistically significant. An RCT on colorectal cancer surgery found that PN with alanyl-glutamine in combination with ω-3 fish oil fat emulsion helped improve patients’ postoperative nutritional status and immune function and reduced the overall incidence of adverse effects.^[26]^ An RCT on head and neck cancer conducted by Sittitrai et al^[27]^ revealed that a perioperative oral glutamine- and fish oil-rich diet not only significantly reduced surgical complications, length of stay, and hospitalization costs, but also improved patients’ nutritional status, including body weight, prealbumin, and transferrin levels. Similar to the above studies, the results of the current study found that the combined use of alanyl-glutamine and ω-3 PUFAs significantly elevated patients’ total protein and Alb levels. Simultaneously, it significantly lowered the levels of CRP, an inflammatory marker, and attenuated the inflammatory response, in addition to reducing the overall incidence of postoperative complications and improving patient prognosis.

However, other studies have reported conflicting results. A meta-analysis of glutamine dipeptide supplementation for elective major surgery by Sandini et al^[28]^ showed that GLN supplementation did not affect the overall incidence of postoperative complications or postoperative infections. Similarly, an RCT by Ma et al^[29]^ suggested that perioperative use of immunomodulatory nutrients such as glutamine and omega-3 fatty acids during the perioperative period for gastric adenocarcinoma or gastrointestinal mesenchymal stromal tumors does not result in a significant change in inflammatory markers, infectious complications, or overall complications in patients.

In our study, the Alb and TP of U1 in the GC group were lower than those of the other 2 groups, but the Alb and TP of U2 were not significantly different from those of the remaining 2 groups, suggesting that the combined use of glutamine and ω-3 PUFAs in the presence of a much lower Alb and TP enhanced the nutritional status of postoperative patients. Additionally, we found that the change in CRP level in the GC group was greater than that in the other 2 groups, suggesting that the combined use of glutamine and ω-3 PUFAs can more effectively reduce the inflammatory response of patients. Concurrently, the length of hospitalization showed a statistically significant difference among the 3 groups (*P* < .05), and GB had the shortest duration of hospitalization compared with GA. This finding may be attributed to the use of ω-3 PUFAs alone or to the limited number of cases. Furthermore, the total incidence of postoperative complications in the GC group was significantly lower than that in the other 2 groups, indicating that the combined use of glutamine and ω-3 PUFAs can more effectively reduce the incidence of postoperative complications in patients and promote rapid recovery. These findings collectively confirm the advantages of the combined therapy.

The retrospective design of this study was a major limitation. However, unless clinically necessary, the treatment protocol and clinical outcomes at our center were implemented without significant deviations. The major difference was in the PN type among the treatment protocols of the 3 groups. Notwithstanding the aforementioned limitations, this study is the first to demonstrate that postoperative administration of alanyl-glutamine and ω-3 PUFAs in PN for patients with gastroduodenal perforation reduces postoperative complications, enhances nutritional status, mitigates inflammatory responses, shortens hospital stays, and improves prognosis. Without doubt, a prospective RCT and a multicenter study with an expanded sample size should be conducted to further evaluate the value of PN with alanyl-glutamine and ω-3 PUFAs in gastroduodenal perforation surgery and to elucidate the mechanisms underlying these beneficial effects.

## Author contributions

**Conceptualization:** Xuanjun Liu, Gan He, Qigang Li.

**Data curation:** Xuanjun Liu, Weixu Mao, Guowei Zhao, Juan Liao.

**Formal analysis:** Juan Liao.

**Funding acquisition:** Xuanjun Liu, Qigang Li.

**Investigation:** Weixu Mao.

**Writing – original draft:** Xuanjun Liu, Weixu Mao, Guowei Zhao, Gan He.

**Writing – review & editing:** Xuanjun Liu, Weixu Mao.

## Supplementary Material


